# A fatal case of acute hepatitis E among pregnant women, Central African Republic

**DOI:** 10.1186/1756-0500-3-103

**Published:** 2010-04-15

**Authors:** Charles M Goumba, Emmanuel R Yandoko-Nakouné, Narcisse P Komas

**Affiliations:** 1 Service de Gynécologie-Obstétrique, Hôpital Communautaire de Bangui, PO Box 1379, Central African Republic; 2 Emerging Diseases Laboratory, Institut Pasteur de Bangui, Avenue de l'Indépendance x Rue Louis Pasteur, PO Box 923, Bangui, Central African Republic; 3 Viral Hepatitis Laboratory, Institut Pasteur de Bangui, Avenue de l'Indépendance x Rue Louis Pasteur, PO Box 923, Bangui, Central African Republic

## Abstract

**Background:**

Hepatitis E virus (HEV) is a major public health problem in developing countries. HEV infection in pregnant women is more common and more often fatal in the third trimester. The mortality rate due to HEV-induced hepatitis is as high as 15-20 per cent. The present study was designed to determine the potential factors responsible for high mortality rate among pregnant women.

**Findings:**

Twenty one pregnant women attended the Maternity Center of Begoua in the Central African Republic during an outbreak of hepatitis E virus between July and October 2002 with symptoms of acute liver disease. Their mean gestational period was 29.9 (SD 8.3 weeks) and they were aged from 15 to 39 years old. The serology IgM showed that seven women (33%) had acute hepatitis E. Among them, one woman, aged 35 and her newborn died after an apparently normal preterm delivery. The 6 remaining young women, age 18 - 22, had preterm deliveries which included three live babies and three stillborn with one macerated.

**Conclusions:**

These results suggest that maternal age, in addition to hormonal, immunological and environmental factors, may be a risk factor for fatal outcome.

## Findings

Hepatitis E virus (HEV) infection, a common cause of water-borne epidemics, is endemic and frequently responsible for acute viral hepatitis in developing countries. On the contrary, in the industrialized world, anti-HEV antibodies are detected in the general population [1] with no significant morbidity. Transmission is usually feco-oral, although person-to-person transmission has also been reported. Usually, an acute HEV infection is self-limiting, shows no evidence of chronic HEV infection in humans, and has a case-fatality rate of less than 0.1%. However, this disease is responsible for high maternal mortality during the third trimester of pregnancy in India while reports from Egypt, Europe and the USA have shown that the course and severity of HEV during pregnancy is no different from that observed in non-pregnant women [2]. No clinical case was reported in the Central African Republic until 2001 although hepatitis E (IgG) antibodies have been regularly detected in the healthy population [3]. The first epidemic cases of HEV in the Central African Republic were reported in 2002 [4] and, since then sporadic epidemic outbreaks of hepatitis E were observed. Currently in the Central African Republic, pregnant women are not routinely screened for HEV antibodies. Here, we report on cases of pregnant women who had been infected by hepatitis E in Bégoua, in the Central African Republic, in 2002. The aim of this report is to bring to the attention of health authorities the damage caused by the hepatitis E virus among pregnant women in the Central African Republic.

From July to October 2002, during an outbreak of viral hepatitis E, 21 pregnant women received prenatal care at the Maternity Center of Bégoua. The age of the patients ranged from 15 to 39 years (mean ± SD is 23.5 ± 6.3). The mean duration of gestation at admission was 29.9 ± 8.3 weeks. Two of the patients were in the first trimester, seven (7) in the second and the twelve (12) remaining in the third trimester.

The study received the ethical approval of the Scientific Committee of the Faculty of Medicine of the University of Bangui. All the patients were evaluated on the basis of history, clinical examination and liver function profile. They had no history of blood exposure or drug consumption. Initial symptoms included jaundice, fever, anorexia, asthenia, hepatomegaly and gastrointestinal pain. Biological tests were conducted on sera obtained from all patients. The levels of alanine aminotransferase were over 10 times the norm. Yellow fever and malaria tests were negative. As it was epidemic in the area at this time, acute hepatitis E was suspected but only confirmed for seven (7) women (33.3%) by ELISA (anti-HEV antibody IgM, Biokit (Sensitivity and specificity at 100%), Spain). Serology for hepatitis B and C could not be performed. Among the fourteen remaining pregnant women, eleven presented anti-HEV antibodies (IgG) showing previous contact with HEV which could not be dated. Only three pregnant women had negative HEV serology and delivered healthy babies: two at term and one at 8 months.

All seven (7) women infected by hepatitis E virus had preterm delivery. Among them three had live babies and three stillborn including one macerated. In the final case, patient 4 (Table 1) was admitted to the Maternity Center of Begoua with a normal temperature and low blood-pressure (90/60 mm Hg). Two days later, she was evacuated to the referral hospital for metrorrhagia. She was delivered of a premature child, who died a few minutes after birth. The mother died 5 hours later of postpartum hemorrhage (Prothrombin time was 31 seconds with the control at 12.2 seconds showing that prothrombin ratio was under 15% and hemoglobin level was at 6 g/L). Although not significant, this outcome differed from the fourteen (14) other women as did the six (6) such outcomes which were observed (Table 1). Several studies have shown that the incidence of HEV infection in pregnancy is high and a significant proportion of pregnant women can progress to fulminant hepatitis, with a high mortality rate during their third trimester [5-7]. In Sudan, a fatality rate of 17.8% was found during an outbreak in Darfur, with a rate of 31.1% among pregnant women [8]. The mechanism of severe liver injury in pregnant women with hepatitis E mortality remains a mystery [1]. Nevertheless, new insights into the pathophysiology of the interaction of hepatitis E and pregnancy suggest the involvement of immunology and host susceptibility factors and their interaction plays a role in disease processes [2].

**Table 1:** Obstetrical, biochemical and serological data of Central African Pregnant Women with acute viral hepatitis E

Patient Order	Age (years)	Gestational Number	Parity	ALAT (IU)	HEV IgM	HEV IgG	Gestational age (in week)	Delivery (baby status)	Weight at birth in gram	Mother status
1	21	III	I	2200	Pos	-	33	Premature (Macerated Stillborn)	2350	Survived
2	19	II	I	1420	Pos	-	28	Premature (alive)	1970	Survived
3	22	IV	II	1130	Pos	-	37	Premature (alive)	2630	Survived
4	35	VI	V	1030	Pos	-	32	Premature (Died few min later)	1400	Died
5	20	I	0	1260	Pos	-	31	Premature (alive)	1600	Survived
6	20	VI	III	980	Pos	-	28	Premature (Stillborn)	NA*	Survived
7	18	II	I	1380	Pos	-	28	Premature (Stillborn)	NA	Survived
8	30	I	0	704	Neg	Pos	22	Premature (Stillborn)	1000	Survived
9	35	IX	VIII	940	Neg	Pos	22	Miscarriage	400	Survived
10	17	II	I	700	Neg	Pos	40	term (alive)	NA	Survived
11	26	IV	III	973	Neg	Pos	28	Premature (alive)	1910	Survived
12	33	VIII	VI	3560	Neg	Pos	19	Miscarriage	NA	Survived
13	19	I	0	950	Neg	Pos	28	Premature (alive)	NA	Survived
14	22	I	0	1130	Neg	Pos	39	term (alive)	NA	Survived
15	24	IV	III	1004	Neg	Pos	36	Premature (alive)	1850	Survived
16	16	I	0	225	Neg	Pos	32	Premature (alive)	1900	Survived
17	30	III	II	110	Neg	Neg	32	Premature (alive)	NA	Survived
18	18	I	0	340	Neg	Neg	41	term (alive)	NA	Survived
19	20	II	I	135	Neg	Pos	32	Premature (alive)	NA	Survived
20	20	I	0	632	Neg	Neg	40	term (alive)	NA	Survived
21	15	I	0	820	Neg	Pos	40	term (alive)	2300	Survived

* Not Available

Our study shows that the differences between these seven women consisted of their gestational number, their parity and their age. Only the 35 year old died five hours after she delivered her baby. All the other pregnant women infected were much younger (18 - 21 years old) even though some had the same gestational number as that of the deceased. This leads to the conclusion that the age of the patient might be another factor, in addition to hormonal and immunological factors, in the mechanism leading to mortality of the pregnant women in their third trimester. Our study is limited by the fact that only seven pregnant women presented acute hepatitis E and only one of them died.

Although several studies have been carried out on the factors that could be involved in the mortality of pregnant women infected by hepatitis E in their third trimester, few were related to the interactions of age/hormonal factors, age/immunological factors and age/environmental factors. It would be interesting to explore this avenue in order to decipher the influence of age on the fatal evolution of this disease among pregnant women.

Since vertical transmission risk was reported for hepatitis E virus [9] the death of the newborn and also the stillborn babies were likely due to the mother-child transmission by the mother who presented an acute hepatitis either during the delivery or during the third trimester of pregnancy [10-12]. It will also be interesting to explore this avenue in order to find better mechanisms for preventing this transmission.

In addition to the observation that a decrease in cellular immunity during pregnancy, coupled with high levels of steroid hormones, may influence hepatitis E viral replication to cause the reported high mortality [13], age may also be a risk factor that may have played a role in the fatal case observed. The mortality rate of hepatitis E is elevated in adults and the conjunction of age, multiparity and pregnancy should be further investigated to confirm this observation. Screening and follow up studies of pregnant women and their newborns for HEV infection are thus important for improving knowledge about the epidemiology (transmission and circulation) and transmission pathway of this virus. More extensive studies should be conducted to characterize the circulating HEV genotypes and to determine the current pathological and risk status in the general population as a whole and in pregnant women in particular in the Central African Republic.

## Competing interests

The authors declare that they have no competing interests.

## Authors' contributions

CMG gynecologist-obstetrician at the Maternity Center of Bégoua. He examined the patients and collected blood samples from each patient. He also followed up all positive anti-IgM HEV antibody pregnant women in the Maternity Center of Bégoua. ERNY participated in the design of the study and carried out the immunoassays. He also participated in interpretation of data. NPK conceived of the study, participated in its design and directed its implementation. He performed the data analysis. He drafted the manuscript.

All authors read and approved the final manuscript

## Consent

Written informed consent was obtained from the patient for publication of this case report and any accompanying images. A copy of the written consent is available for review by the Editor-in-Chief of this journal.
